# Immunologic Diagnosis of Endemic Mycoses

**DOI:** 10.3390/jof8100993

**Published:** 2022-09-22

**Authors:** Rodrigo Almeida-Paes, Andrea Reis Bernardes-Engemann, Beatriz da Silva Motta, Claudia Vera Pizzini, Marcos de Abreu Almeida, Mauro de Medeiros Muniz, Renata Alves Barcelos Dias, Rosely Maria Zancopé-Oliveira

**Affiliations:** Laboratório de Micologia, Instituto Nacional de Infectologia Evandro Chagas, Fundação Oswaldo Cruz, Rio de Janeiro 21040-900, Brazil

**Keywords:** antibody, antigen, blastomycosis, coccidioidomycosis, histoplasmosis, paracoccidioidomycosis, cryptococcosis, sporotrichosis, talaromycosis, emergomycosis

## Abstract

The endemic mycoses blastomycosis, coccidioidomycosis, histoplasmosis, paracoccidioidomycosis, cryptococcosis, sporotrichosis, talaromycosis, adiaspiromycosis, and emergomycosis are mostly caused by geographically limited thermally dimorphic fungi (except for cryptococcosis), and their diagnoses can be challenging. Usual laboratory methods involved in endemic mycoses diagnosis include microscopic examination and culture of biological samples; however, serologic, histopathologic, and molecular techniques have been implemented in the last few years for the diagnosis of these mycoses since the recovery and identification of their etiologic agents is time-consuming and lacks in sensitivity. In this review, we focus on the immunologic diagnostic methods related to antibody and antigen detection since their evidence is presumptive diagnosis, and in some mycoses, such as cryptococcosis, it is definitive diagnosis.

## 1. Introduction

Endemic mycoses are mostly caused by thermally dimorphic fungi that present a limited geographic distribution, occupying specific ecologic niches in the environment, and can cause both primary or opportunistic diseases [1]. In addition, endemic mycoses are recognized as significant causes of morbidity and mortality predominantly in HIV/AIDS and other immunosuppressive conditions, including immunosuppressant drugs [2]. The most common endemic mycoses are blastomycosis, coccidioidomycosis, histoplasmosis, paracoccidioidomycosis, cryptococcosis, sporotrichosis, and, more recently, talaromycosis, adiaspiromycosis, and emergomycosis, considered emerging endemic mycoses [3]. In recent years, the number of endemic mycoses cases has risen worldwide [1]. In addition, there are significant variations in their geography, clinical presentation, roentgen manifestations, analytic diagnostic methods, and therapeutics. Their proper control involves recognition of risk factors (e.g., putative environmental sources of fungal exposure in endemic areas), correct diagnostic procedures, and therapeutic management [4].

The diagnosis of endemic mycoses is difficult to achieve. Precise laboratory data evaluation is necessary to guarantee appropriate therapy for patients. Although the manifestations of endemic mycoses are well defined, their diagnosis cannot be centered solely on patient’s clinical data, since the signs and symptoms of endemic mycoses overlap among them and with other infectious diseases [3].

The association of clinical, epidemiological, and laboratorial data typically diagnoses endemic mycoses. To corroborate the diagnosis, laboratorial tests must be performed. The usual laboratory tests involved in endemic mycoses diagnosis comprise the microscopic examination and culture of several types of biological samples. The microscopic aspect of the agents is often indicative in the case of endemic mycosis, but considerable laboratory expertise is necessary and sensitivity of these methods is variable. Culture from possibly involved sites remains the diagnostic gold standard method, despite longtime fungal growth (up to six weeks in some cases) and the need for biosafety level 3 facilities for handling some agents in the laboratory [5].

Currently, there are further diagnostic tools available for diagnosis of endemic mycoses to complement culture and direct examination [6]. These complementary methods have fast turnaround time and satisfactory efficiency. Different immunologic techniques concerning antibody and antigen detection have been developed to aid in the diagnosis of endemic mycoses (Table 1). Several antigenic preparations have been used in these tests, from crude to purified antigens, as well as recombinant proteins and synthetic peptides. However, the latter are not used in the validated assays for routine mycology laboratories. As mentioned before, serologic evidence of these infections is valuable due to the time-consuming nature and low sensitivity of gold-standard methods. In addition to antigen and antibody detection methods, intradermal skin tests were largely employed in the last century [7,8,9], but their current use for diagnostic purposes in medical mycology is severely limited, due to the lack of standardized antigens, advances in antibody and antigen detection methods, and biosafety requirements to perform the skin tests. Molecular tools of dimorphic fungal DNA detection in biological samples are also being standardized and validated in numerous laboratories to simplify diagnosis. Unfortunately, although promising and useful, non-culture diagnostic tools are not accessible in most low-income countries.

The next sections will focus on the immunologic diagnostic applications of the endemic mycoses. A number of well-established tools will be discussed and reviewed. Furthermore, we will summarize the progress in the development of new serologic tests and their relative advantages.

## 2. Blastomycosis

Blastomycosis is a fungal infection of humans and mammals caused by dimorphic fungi of the genus *Blastomyces*. Its major etiologic agents include *Blastomyces dermatitidis*, *Blastomyces gilchristii*, and *Blastomyces percursus*. Blastomycosis frequently affects immunocompetent individuals; however, immunocompromised patients are more likely to present severe disease [10].

Direct visualization of *Blastomyces* spp. in biological samples can provide fast diagnosis, making it possible to initiate proper antifungal therapy. Correct visualization of fungal elements is sometimes difficult to achieve by hematoxylin-eosin (H&E) staining, thus the periodic acid–Schiff or methenamine silver stains are recommended. Potassium hydroxyde or calcofluor white direct examination are valuable for specimens from the respiratory tract [3]. As for other endemic mycoses, culture is the gold standard diagnostic method. Sabouraud dextrose agar cultures usually demonstrate mold *Blastomyces* sp. colonies within weeks to months.

### 2.1. Antibody Detection

The complement fixation reaction was used as the first immunological test for blastomycosis diagnosis using the yeast-form derived antigen, but its sensitivity (57%) and specificity (30%) were low. After the introduction of the immunodiffusion test in 1973 using *B. dermatitidis* specific “A” antigen, a culture filtrate [11], the efficiency of the test was enhanced [12,13]. The evaluation of the purified “A” antigen, instead of the crude yeast antigen, and its application in the complement fixation test resulted in high specificity, even though sensitivity was 62% [14]. Afterwards, the value of purified “A” antigen was evaluated in an enzyme-linked immunosorbent assay (ELISA), revealing 92% sensitivity and 84% specificity when comparing its diagnostic efficacy to blastomycosis with complement fixation and immunodiffusion tests as gold standards [15].

The reagents commercially available for *Blastomyces* antibody detection have been used for several years; nevertheless, they are currently judged unsatisfactory for blastomycosis diagnosis. Immunodiffusion performed with the purified *B. dermatitidis* “A” antigen was shown to be more efficient than complement fixation. Immunodiffusion precipitation bands are specific for blastomycosis, but their absence does not rule out diagnosis, since the test sensitivity ranges from 65% to 80% [14,16]. Undoubtedly, EIA for diagnosis of blastomycosis developed for antibody detection is more sensitive than immunodiffusion; however, it is less specific [15]. The EIA provided a noteworthy advance in immunologic testing for blastomycosis and could be performed during outbreaks as an epidemiological tool to detect acute *B. dermatitidis* infection; titers higher than or equal to 1:32 powerfully support blastomycosis diagnosis, while titers of 1:8 or 1:16 are only indicative of blastomycosis [15].

Linder and Kauffman (2020) summarize the main points of standard immunodiffusion and complement fixation assays, which are valuable for histoplasmosis diagnosis, but have not showed satisfactory sensitivity and specificity to support blastomycosis diagnosis [17]. Further improvements aiming to measure antibodies to the WI-1 (BAD-1) antigen, an important fungal adhesin, look like they have better sensitivity [18]. An EIA directed to the WI-1 antigen presented superior efficiency when compared to previous tests [19]. The report of sensitivity and specificity was 88% and 99%, respectively.

Klein and Jones (1990) developed a radioimmunoassay (RIA) to detect antibodies anti- WI-1 (also known as BAD-1) antigen, demonstrating positivity in 85% of blastomycosis patients and just 3% of patients with other mycoses, proving to be superior to the EIA using the “A” antigen (58% positivity) [20]. Several studies validate the initial results [21,22,23], but up to now, no commercially available kit for clinical testing using this method has been manufactured.

### 2.2. Antigen Detection

The quantitative Sandwich EIA is produced by the MVista^®^, and is applied for the antigen detection test for the diagnosis of blastomycosis. A galactomannan of the cell wall of *B. dermatitidis* is the target of the MVista^®^ enzyme immunoassay. The test is useful for diagnosis and monitoring of disease and patient management. Cross-reactions are seen with histoplasmosis, paracoccidioidomycosis, talaromycosis, infrequently in coccidioidomycosis, uncommonly in aspergillosis, and probably in patients with sporotrichosis [24,25]. This assay can be performed with urine, serum, bronchoalveolar lavage (BAL), and cerebrospinal fluid (CSF) samples [26,27,28]. The majority of the data about efficiency are stated for urine, in which sensitivity varies from 76% to 90% in several studies [24,26,27,29]. Sensitivity of the sandwich EIA is lower in serum, ranging from 56% to 82% [25,27,28]. Pretreatment with ethylenediaminetetraacetic acid and boiling to 104 °C to dissociate immune complexes improved antigen detection in serum samples, increasing antigenemia from 35.7% to 57.1% [25]. Sensitivity of antigen detection in BAL and CSF is unknown, but has been informed to help diagnosis of particular cases [26].

Several fungi share galactomannan antigens, and therefore, the specificity for a particular genus is usually not high enough. For instance, the cross-reactivity of *Histoplasma* and *Blastomyces* antigens has ranged between 93 and 96% using this EIA assay [24,25]. Although several patients with blastomycosis present false positive results for *Aspergillus* galactomannan, no patient with aspergillosis presented a positive antigen assay for *B. dermatitidis* [30].

Some research groups have stated that the antigen follow-up in urine can be helpful for checking the resolution or progression of blastomycosis [28,31,32]. The *Blastomyces* urine antigen detection could also be valuable to follow up the therapeutic response, since clearance of antigen correlates well with the patient’s recovery [28]. In addition, this test appears to diagnose blastomycosis regardless of its etiological agent [33].

## 3. Coccidioidomycosis

The two cryptic and dimorphic fungi *Coccidioides immitis* and *Coccidioides posadasii* cause coccidioidomycosis. The former species occurs in the Central Valley of California (San Joaquin Valley), but has now been detected as far north as east of Washington [34]. The latter species is regularly found in Arizona, Texas, Utah, Mexico, and Central and South America [35]. The at-risk individuals to be infected with these fungi include archeologists, laboratory staff handling the coccidioidomycosis agents, and visitors to endemic areas [34]. Coccidioidomycosis is often asymptomatic or occurs as a respiratory syndrome with undistinguishable, self-limiting symptoms. On the other hand, depending on the patient’s immunity, symptomatic, disseminated, and severe infections may occur [36].

Microscopic examination is fast and effective for coccidioidomycosis diagnosis, and culture confirms the species. Spherules of 20 to 70 μm in diameter, or even larger, with a double membrane containing endospores (2–5 μm), are observed in biological samples by microscopy [4]. Histopathologic tests show tuberculoid and mixed granulomas with spherules of different sizes [37]. The intradermal coccidioidin skin test and antibody detection by complement fixation are the most used immunological diagnostic tools for coccidioidomycosis. Both tests are useful for prognosis [4].

Among the immunological methods on hand for the endemic mycoses, those for coccidioidomycosis have been the most trustworthy. The following tests have been used for diagnosis: complement fixation and precipitin reactions in numerous versions, i.e., tube precipitin, immunodiffusion tube precipitin, immunodiffusion complement fixation, and quantitative immunodiffusion complement fixation, agar gel precipitin-inhibition test, and counterimmunoelectrophoresis; latex particle agglutination; fluorescent antibody; RIA, ELISA, and mycoarray.

### 3.1. Antibody Detection

Smith and collaborators established the tube precipitin and complement fixation tests [38,39]. They observed positive tube precipitin reactivity within weeks of infection. On the other hand, complement fixation positivity occurred later, usually within 2 to 3 months after fungal exposure. Moreover, complement fixation titers might increase if coccidioidomycosis was not under control.

The appearance of immunodiffusion complement fixation and sporadic immunodiffusion tube precipitin bands between a serum sample and the *Coccidioides* antigen is probable evidence of coccidioidomycosis, active or recently acquired, and a negative test does not exclude mycosis [40].

Latex agglutination and complement fixation assays may offer relevant additional data about the patient status [41]. The complement fixation assay has high sensitivity; however, its performance is complex and laborious. In addition, the complement fixation assay has low specificity due to cross-reactivity that may occur with antibodies recognizing common fungal carbohydrate moieties. The immunodiffusion assay is more specific, and complement fixation is more sensitive [42].

The use of complement fixation and immunodiffusion tests for coccidioidomycosis diagnosis is well established with crude antigen samples, known as coccidioidins, which include the reactive complement fixation antigen, as well as several important molecules. The production of purified antigens could improve the effectiveness of immunological tests. The *C. posadasii* Silveira antigenic preparation has a protein with a 110 kDa molecular weight that migrated at 48 kDa when fractionated under heated and reducing SDS-PAGE conditions. The use of recombinant chitinase antigen in conventional complement fixation and immunodiffusion complement fixation has been reported [43].

Several tests for antibody detection in coccidioidomycosis have been reported, demonstrating very relevant outcomes [44]. In an effort to improve sensitivity of immunologic diagnosis, Meridian Diagnostics (Cincinnati, OH, USA) established an ELISA (Premier Coccidioides EIA kit) for IgM and IgG antibody detection against *Coccidioides* spp. as well as for the detection of antibodies against a 33 kDa cell-wall purified antigenic molecule from immature *C. immitis* spherules [45].

The method “mycoarray” is composed of three antigen extracts (histoplasmin, coccidioidin, and *Coccidioides* “TP”) for antibody detection. Microarray slides are probed with coccidioidomycosis and histoplasmosis serum samples from patients or from healthy individuals and the detection of immunocomplexes is carried out by indirect immunofluorescence. In concordance with clinical and mycological diagnosis, the “mycoarray” could distinguish between these two mycoses and clearly discriminate between IgM and IgG antibody reactivity. After a proper validation and with its employ as a large-scale array, the “mycoarray” could be applied to help clinicians provide coccidioidomycosis diagnosis [46].

The immunologic response of an in-house antigen preparation, obtained from a *C. posadasii* strain isolated in Ceará, northeastern Brazil, was evaluated by immunodiffusion and Western blot. In addition, its biochemical characterization was performed. Two immunoreactive proteins were characterized as a β-glucosidase and a glutamine synthetase after analyses of their respective N-terminal sites. This in-house *Coccidioides* preparation could be promising as a fast and low-cost diagnostic method [47]. This study, however, does not contain conclusions on immunologic data.

Other studies directed to antigenic fractions recognized by anti-*Coccidioides* antibodies in serum samples from coccidioidomycosis patients were carried out more recently, and the obtained proteins were analyzed by homology to species-specific *Coccidioides* peptides. A *C. immitis* specific peptide was selected from the “GPI anchored serine-threonine rich protein OS” that recognized both *C. immitis* and *C. posadasii*. These peptides can be employed in diagnostic reagents, immunobiologicals, and antifungal drugs [48].

### 3.2. Antigen Detection

Antibody detection has been used as the principal coccidioidomycosis diagnostic method, but it presents some weaknesses. Kassis and collaborators evaluated in retrospect the efficiency of antigen and antibody detection in 158 coccidioidomycosis cases and 487 controls. The sensitivity of combining antigen and immunodiffusion antibody detection was 93.0%. The sensitivity of antigen detection in urine and serum samples was 55% in proven coccidioidomycosis and 59% in probable coccidioidomycosis, 79% in disseminated coccidioidomycosis, 42% in pulmonary cases, 75% in immunocompromised individuals, and 40% in immunocompetent individuals. Specificity was 99% for antigen detection and 96% for antibody detection using the immunodiffusion method. Accuracy was determined as 95% for immunodiffusion antibody and antigen detection, 94% for immunodiffusion antibody alone, and 89% for pathology or culture [49]. These findings supported the detection of antibodies and antigens to diagnose progressive coccidioidomycosis. An incorrect diagnosis would occur if antigen detection was not carried out.

An inhibition ELISA was developed to detect and quantify *Coccidioides* chitinase-1 (CTS1) in human sera using a monoclonal antibody reactive for this protein. CTS1 was quantified in commercial antigenic reagents using recombinant CTS1 as the standard. The amounts of CTS1 in diagnostic commercial antigens from distinct suppliers varied. CTS1 antigenemia was observed in 87% of patients with proven or probable coccidioidomycosis. Specificity was determined to be 97% using sera from Phoenix, Arizona residents who did not have coccidioidomycosis. Levels of CTS1 could be associated with low- and high-titer serology from individuals with proven coccidioidomycosis diagnosis [50]. The CTS1 antigen detection assay has the possibility of similar or better performance than other immunologic assays as well as the distinct advantage of a direct measurement of fungal antigen concentrations in blood. Even though further studies are necessary to specify the real role of this assay in mycology laboratories, it could be used as a convenient instrument for difficult-to-diagnose cases.

## 4. Histoplasmosis

Histoplasmosis is a systemic mycosis caused by the dimorphic fungus *H. capsulatum* and is the major endemic mycosis in the United States and in a large part of Latin America [51,52]. In Africa, in addition to classical histoplasmosis, African histoplasmosis, caused by *Histoplasma duboisii*, is also endemic [53]. *H. capsulatum* is a primary fungus, and can cause serious infection in immunocompetent patients, and depending on the patient’s immunity, symptomatic, disseminated, and severe infections may occur.

Histoplasmosis diagnosis is a challenge and often requires a multifactorial approach. Identification of *H. capsulatum* in biological samples by direct microscopy and/or culture is still the gold standard for diagnosis [54,55]. However, these tests still have some limitations: (i) the low sensitivity, which varies according to the clinical form of HPM; (ii) the lengthy cultivation of the fungus, taking 4 to 6 weeks and still requiring conversion to the yeast-like form; (iii) the need for a biosafety level 3 facility for handling *H. capsulatum* [56]. Thus, immunologic methods of antibody and antigen detection are options for the presumptive diagnosis of histoplasmosis using serum, plasma, CSF, and urine as clinical specimens [55].

### 4.1. Antibody Detection

The time required for anti-*H. capsulatum* antibody development is two to six weeks after fungal exposure [54]. Some of the available serological tests for detecting anti-*Histoplasma* antibodies are immunodiffusion, complement fixation, latex agglutination, ELISA, and Western blot. The two most used methods until recently for antibody detection in biological samples are immunodiffusion and complement fixation, usually performed in reference laboratories due to the convenience, availability, and precision of these assays [57,58].

Immunodiffusion using the histoplasmin antigen (HMIN), an antigenic preparation obtained from the mycelium-form cultures of *H. capsulatum*, detects the presence of antibodies through the appearance of H and M precipitins. The H precipitin usually co-exists with the M precipitin; however, the latter often occurs alone. Anti-M antibodies are triggered in acute or chronic histoplasmosis and in some individuals who have undertaken the histoplasmin skin test. In addition, the M precipitin can persist for years [59]. H precipitin usually appears after the M precipitin and is suggestive of chronic or severe histoplasmosis. Anti-H antibodies are rarely observed in the routine diagnosis (20%), but, when detected, corroborate with a histoplasmosis diagnosis [55]. Although the specificity of the test is 100%, the sensitivity varies from 70 to 95%, according to the histoplasmosis clinical form [58]. The detection of both precipitins (H and M) is thought to be decisive for the histoplasmosis diagnosis, although the mycosis condition requires an evaluation of the patient [57].

The complement fixation test detects antibodies against the yeast and mycelial phase histoplasmin. Although often more sensitive (72–95%) than immunodiffusion, depending on the antigen used, complement fixation is less specific and may present cross-reactivity with serum samples from patients with *B. dermatitides*, *C. immitis*, *Paracoccidioides brasiliensis*, and *Candida* sp. infections [58,60]. As for the interpretation of the results, titers equal or higher than 1:32 or four times increase in antibody titers of acute and convalescent disease indicate active infection. Titers of 1:8 generally suggest prior *H. capsulatum* exposure [61].

Latex agglutination tests were developed for the diagnosis of histoplasmosis, and despite some reports that this test is more sensitive than complement fixation using histoplasmin as the antigen, the specificity of the test was compromised [62]. False positive results may occur in patients with another infectious disease, e.g., tuberculosis [63], and with inflammatory diseases such as rheumatoid arthritis [64].

It has already been demonstrated that immunoassays such as ELISA [65] and Western blot [66] have higher sensitivity than immunodiffusion and complement fixation in the detection of antibodies. Several ELISA protocols for detecting antibodies anti-*Histoplasma* using different antigenic preparations have been described; however, most of them are developed for an *in house* use and present varied degrees of sensitivity and specificity [57,58]. For instance, an ELISA assay with an *H. capsulatum* yeast cell antigenic preparation showed an 86% sensitivity and a specificity of 91% in patients with acute pulmonary histoplasmosis detecting human IgG, but when detecting IgM, the sensitivity decreased to 66% and the specificity rose to 100% [67]. The ELISA test with a proprietary MVista^®^ *Histoplasma* antigen used for evaluating the acute pulmonary form of histoplasmosis detected IgG antibodies in 87%, IgM antibodies in 67%, and IgG and/or IgM antibodies in 89% of patients with this clinical form of histoplasmosis [68]. Another indirect ELISA using purified and deglycosylated histoplasmin was 92% sensitive and 96% specific [65]. The same assay was evaluated for different clinical forms of histoplasmosis, yielding positive results in 100% of acute patients, 90% of chronic patients, 89% of disseminated infection in individuals without HIV infection, 86% of disseminated disease in people living with HIV/AIDS (PLWHA), and 100% of mediastinal histoplasmosis patients [69]. More recently, an ELISA using a similar antigen, deglycosylated extracellular released antigen, showed 72% and 98% sensitivity and specificity, respectively. In this study, 100% from the patients with acute form, 50% with chronic form, and 66.67% with disseminated form, respectively, were positive [70].

A Western blot test using purified and deglycosylated histoplasmin was developed, evaluated, and validated, showing sensitivity of 94.9% and specificity of 94.1%. In addition to being simpler and faster, strips sensitized with the purified and deglycosylated histoplasmin antigen were shown to be viable for use for at least five years [66,71,72], and can also be applied with high sensitivity even in PLWHA [73].

### 4.2. Antigen Detection

Antigen detection tests are particularly valuable in the diagnosis of disseminated histoplasmosis in PLWHA whose antibody levels are low or inexistent. They provide high sensitivity for the diagnosis of histoplasmosis, and are now incorporated in the World Health Organization (WHO) Essential Diagnostics List [74]. During histoplasmosis, the antigen can be liberated from fungal cells and detected in biological samples such as serum, urine, CSF, BAL, and pleural fluid [58]. Antigen detection assays can also be applied in the histoplasmosis follow-up. However, a limitation to these tests is the substantial cross-reactivity with other mycoses, including paracoccidioidomycosis, blastomycosis, talaromycosis, coccidioidomycosis, and aspergillosis [55].

The RIA method was the first test described for the recognition of *H. capsulatum* antigens. Based on the detection of *H. capsulatum* polysaccharide antigen in urine and serum of patients, it proved to be effective to diagnose this infection, especially in individuals with disseminated histoplasmosis. Since its development in 1986, and with the improvement of the technique, there has been an increase in detection levels of antigens, demonstrating a sensitivity of 96.7% in urine and 78.7% in sera from PLWHA and disseminated histoplasmosis patients [75,76]. False positive results may occur in individuals with blastomycosis or paracoccidioidomycosis [77] and this test has been performed in an EIA format to avoid exposing workers to radioactivity [78].

ELISA, in its various protocols, is another method using for *Histoplasma* antigen detection [79,80,81,82]. A quantitative ELISA assay was developed, and the concentrations of *H. capsulatum* galactomannan antigen were established by comparing them to a standard curve constructed with a purified galactomannan from the *H. capsulatum* yeast-like form. Serum and urine samples were tested, showing a sensitivity of 92.3% in serum samples and 100% in urine from the disseminated histoplasmosis cases. Cross-reactions were detected in 70% of patients with other endemic mycoses (blastomycosis, paracoccidioidomycosis, coccidioidomycosis, and talaromycosis) [83]. The same test, MVista *Histoplasma* antigen enzyme assay, was changed to allow the quantitative determination of antigen in BAL and this method was compared to culture and cytopathology. Antigen was detected in BAL in 93% of patients with histoplasmosis, and culture and cytopathology both showed 48% sensitivity. Combining antigen detection and cytopathology in BAL, both rapid diagnostic tools, the sensitivity was 96.8%. Thus, BAL antigen detection complements antigenemia and antigenuria as a diagnostic tool for histoplasmosis. However, cross-reactivity is observed in patients with blastomycosis (80%) [84].

A multicenter study described by Hage and collaborators [85] evaluated the sensitivity and specificity of the ELISA for the detection of MVista^®^ *Histoplasma* antigen (MiraVista Dianostics) in different clinical forms. A sensitivity of 91.8% was found in urine from individuals with disseminated histoplasmosis, 83.3% with acute histoplasmosis, 30.4% with the subacute form, and 87.5% with the chronic pulmonary form. In serum samples, the test showed a sensitivity of 100% in cases of disseminated histoplasmosis. Specificity was 99% between individuals with non-fungal infections and healthy individuals; however, 90% of patients with blastomycosis presented cross-reactivity.

Another study evaluated two commercial kits for histoplasmosis diagnosis in immunocompromised individuals. The FDA-cleared in vitro diagnostic assay kit (Alpha *Histoplasma* Antigen EIA) uses a rabbit polyclonal antibody or a monoclonal anti-*Histoplasma* galactomannan antibody (Immuno Mycologics – IMMY, Norman, OK, USA). The assay using the monoclonal antibody presented higher sensitivity (90.5%) and specificity (96.3%) than the test performed with the polyclonal antibody (61.9 and 79.3%) [86]. More recently, an ELISA for the detection of *Histoplasma* antigenuria, developed by Optimum Imaging Diagnostics, was studied, presenting 92% sensitivity. However, false positive results occurred in 68% of samples tested [87].

In the search for rapid tests for the diagnosis of histoplasmosis, a lateral flow assay to detect *Histoplasma* antigenemia settled by MiraVista Diagnostics was studied in three populations: PLWHA with proven histoplasmosis, PLWHA with other infectious diseases, and people without HIV. The test sensitivity was 96% when read visually and 92% when an automated reader was used and the specificities were 90% and 94% for the same conditions [88]. Afterwards, a validation study was carried out on the MVista^®^ Diagnostics *Histoplasma* urine antigen lateral flow assay for antigen detection, and was associated with the MVista^®^ *Histoplasma* Ag quantitative ELISA. The sensitivity of both tests was 96%; however, the specificity was 96% and 77% for LFA and ELISA, respectively [89].

### 4.3. African Histoplasmosis

Most cases of African histoplasmosis reported up to now were diagnosed by culture and histology. Serological tests to diagnose histoplasmosis have been applied in just a few African countries (Benin, Chad Republic, Egypt, Republic of Congo, South Africa, Tanzania, and Uganda). In four cases, the serological test was carried out outside Africa [90,91,92].

Diagnosis of histoplasmosis in Africa is currently attainable using traditional mycological methods (culture or histopathology). Antigen detection, although very sensitive, is not available in most of Africa [91]. According to Cipriano and collaborators, antigen detection in serum and urine has only been developed for *H. capsulatum* [93].

Immunodiffusion tests using histoplasmin produced with *H. duboisii* and *H. capsulatum* strains were used to investigate the presence of antibodies in inhabitants around a natural focus of *H. duboisii* [94]. In a case report of suspected disseminated histoplasmosis by *H. duboisii* in a child from the Chad Republic, cultures could not be performed due to the lack of laboratory infrastructure, but immunodiffusion tests with soluble antigens of *H. capsulatum* and *H. duboisii* were performed and precipitins were observed against both antigens [90].

A recent literature review about African histoplasmosis in the Republic of Congo, with the majority of cases of histoplasmosis reported in Africa, demonstrated fifty-four cases of African histoplasmosis, and only one of them was diagnosed by *Histoplasma* antigen, which was tested in France, in an HIV-positive woman originating from the Republic of Congo [92].

## 5. Paracoccidioidomycosis

Paracoccidioidomycosis, a neglected tropical disease recognized by the WHO [95], has the fungi from the genus *Paracoccidioides* as etiologic agents, *Paracoccidioides brasiliensis* being the most common species, followed by *Paracoccidioides lutzii*. The other cryptic species *P. americana*, *P. restrepiensis*, and *P. venezuelensis* have also been reported. All species are widespread in Latin America, from Mexico to Argentina, being more frequent in Brazil [96].

Paracoccidioidomycosis diagnosis is typically achieved by the association of clinical, epidemiological, and laboratorial information [97]. Substantial progress has occurred in non-culture-based methods employed in the diagnosis of this systemic mycosis, with the development of a diversity of techniques for antibody, antigen, or nucleic acid detection. Immunologic techniques are typically simpler than mycological traditional methods, e.g., culture, and are extremely helpful in the diagnosis and follow-up of patients infected with *Paracoccidioides* spp. These methods underwent substantial advances in the past years as a result of the development of original detection methods and identification of pertinent *Paracoccidioides* antigens [96].

### 5.1. Antibody Detection

Over the years, several groups have evaluated serological techniques for the diagnosis of paracoccidioidomycosis, which provides a presumptive diagnosis and therapeutic follow-up. Despite the variety of assays proposed during years of study, the immunodiffusion test, described by Ouchterlony [98], is employed as the gold standard in the serological diagnosis of paracoccidioidomycosis. Currently, immunodiffusion, counterimmunoelectrophoresis, ELISA, and Western blot are the immunologic tests offered by different reference laboratories [96,97]. The tests employ similar and adequate antigens, presenting sensitivity values ranging from 80% to 95% [96]. The detection of seric anti-*Paracoccidioides* spp. antibodies involves a fungal antigenic preparation, which needs to have satisfactory reactivity in the chosen immunologic test format. This reactivity needs to involve patients infected with either *P. brasiliensis* or *P. lutzii*, in addition to being low in serum samples from patients with other mycoses [99]. The glycoprotein gp43 is the major antigen of *Paracoccidioides* spp., but its production is variable among different fungal species, especially in *P. lutzii*, whose gp43 is released in lower quantities and may present with an altered molecular organization [100].

Negative results in serological testing of patients with confirmed mycological paracoccidioidomycosis are described. Failure in the detection of anti-*Paracoccidioides* antibodies has been associated with issues either related to the methods or to the immunologic condition of the patient. In the last few years, this has been clarified by the perception that *P. lutzii* and *P. brasiliensis* have different antigenic profiles and, consequently, may drive different humoral immune responses in the host [101].

The literature about the immunologic diagnosis of paracoccidioidomycosis is wide and diverse. A variety of serological methods have proven suitable for proper diagnosis in adequate time [102]. For a detailed summary of works in this subject up to 2016, the review of Silva is recommended [103]. Here, we will prioritize the most used tests in the paracoccidioidomycosis presumptive diagnosis.

Double immunodiffusion has been the most used method for the primary diagnosis of individuals with paracoccidioidomycosis [97]. This test has high efficiency, which may range from 65 to 100%, depending on the antigenic preparation employed [104]. The test performance is not affected by HIV-driven immunosuppression [105]. For many years, different antigens have been used for paracoccidioidomycosis immunologic diagnosis, and various antigens lack a standardized preparation from one laboratory to another. The glycoprotein gp43 is considered the most important *P. brasiliensis* antigen. Patients affected by severe paracoccidioidomycosis present high amounts of anti-gp43 antibodies, therefore a strong and long-lasting humoral response to this antigen is usually seen in *P. brasiliensis* infected patients [96]. Actually, around 90% of paracoccidioidomycosis patients can be easily diagnosed by gp43-based immunodiffusion [106]. Some laboratories noticed that false negative results can occur in some cases [96,97,107,108]. Two possibilities could be associated with this situation: (i) immunosuppression of the patient, with insufficient precipitating antibodies in immunodiffusion; (ii) the presence of IgG asymmetric antibodies with a structure predominantly based on the mannose-rich oligosaccharide part connected to the Fc moiety of just one of the Fab arms of the antibody, which are functionally univalent and, therefore, non-precipitating [107]. Additionally, the lack of reactivity on serum samples from patients with *P. brasiliensis* may be associated with the production of low-avidity IgG2 antibodies that bind carbohydrate epitopes [109]. During efficacious treatment, the serum antibody levels detected by immunodiffusion decrease progressively until becoming negative [96], thereby constituting the most useful test for paracoccidioidomycosis cure control [97].

An additional method used in the early diagnosis of paracoccidioidomycosis is counterimmunoelectrophoresis. The time to obtain results of immunodiffusion and counterimmunoelectrophoresis is virtually the same [107]. In addition, counterimmunoelectrophoresis has a sensitivity equal to or slightly higher than immunodiffusion [110]. However, this test is more expensive and is not accessible in several laboratories from endemic areas [107]. Other precipitation techniques, such as immunoelectrophoresis and immunoelectrophoresis-immunodiffusion, are less specific than immunodiffusion, and are also more expensive [111,112]. Therefore, they are usually applied more in research studies than diagnostic tests [113].

Several latex agglutination tests have been described [103]. They have lower specificity and sensitivity, but are faster and are simple to be carried out. For instance, Santos and collaborators described a latex agglutination test with high sensitivity and specificity to detect the anti-gp43 antibody and gp43 antigen when employing latex particles linked to the purified gp43 and anti-gp43 monoclonal antibody [114].

ELISA has been extensively employed for the detection of antibodies to *Paracoccidioides* spp. in biological samples. Different research groups use a variety of antigens in ELISA tests because partially purified crude antigens and purified proteins, such as gp43, normally present high sensitivity but do not have high specificity with certainty [103]. Recombinant antigens, such as r*Pb*27 and r*Pb*40, can be used as well [115]. After some years, a standardization of a yeast filtrate as an antigen provided an increase of sensitivity and specificity [116]. Capture ELISA uses adsorbed monoclonal antibodies directed to gp43 on the plate, and represents progress in the detection of specific antibodies [107]. The standard ELISA is an excellent method for the detection of humoral immune responses in paracoccidioidomycosis patients for laboratories with medium infrastructure [103]. In addition, antibody responses evaluated by this method differ according to the clinical form of the disease. Patients with acute paracoccidioidomycosis present higher IgG titers, while patients with chronic paracoccidioidomycosis have higher IgA production [117].

Western blot has been used to recognize *Paracoccidioides* antigens that react with antibodies in serum samples. Mendes-Giannini and collaborators were pioneers in the development of this methodology for paracoccidioidomycosis diagnosis [118,119], being followed by several other groups [120,121,122,123,124]. The benefit of Western blot in relation to routine immunologic tests is a fast diagnosis of some patients, before complement fixation and immunodiffusion detect seroconversion, in addition to high efficiency [97].

Dot-ELISA has been recognized as a quick, versatile, and effortless test based on the principle of EIA, for the detection of many protozoan, virus, and fungal infections [125]. Dot-ELISA use in paracoccidioidomycosis diagnosis was hitherto presented by three research groups, without fundamental differences in efficiency [126,127,128]. In fact, Dot-ELISA was a particularly innovative tool as an immunologic screening technique, due to its high sensitivity (91%) and specificity (95%) [102]. Furthermore, Dot-ELISA could be performed by laboratories with little infrastructure or even in field work.

### 5.2. Antigen Detection

Although antigen detection would have crucial benefits over antibody detection in paracoccidioidomycosis diagnosis, especially in immunocompromised patients [129], a large scope for enhancement in antigen detection remains, as the last relevant contribution in this field was provided in 2011. Different assays of antigen detection were described, involving different ELISA formats, antigenic targets, and clinical samples, e.g., serum, urine, BAL, and CSF [130,131,132]. The majority of them, however, showed low sensitivity [107].

## 6. Cryptococcosis

Cryptococcosis is a mycosis of worldwide significance, involving both immunocompromised and immunocompetent patients. Traditionally, *Cryptococcus neoformans* and *Cryptococcus gattii* have been the major agents of cryptococcosis [133]. These two fungi share numerous similarities, but diverge in endemic areas, epidemiology, and clinical presentation [134]. *C. neoformans* occurs worldwide. *C. gattii* has been identified for several years, especially in tropical parts of Australia, Asia, Africa, and the Americas, but after the outbreak in Vancouver Island, *C. gattii* has gained prominence, with cases of *Cryptococcus gattii* in mammals from other areas of southwestern Canada and the northwestern United States [135].

The diagnosis of cryptococcosis, regardless of the causative species (*C. neoformans* or *C. gattii*), is classically performed using microscopy, immunologic methods, or microbiologic methods. India ink direct examination is a low-cost and fast method to detect *Cryptococcus* spp. in CSF and other body fluids. The stain fills the background field, but is not taken up by the *Cryptococcus* capsule, forming a bright light halo under regular microscopy. Though highly specific, the sensitivity of India ink microscopy (around 86%) is user-dependent and notably inferior in early infection, when the fungal burden is minor [136]. Therefore, its use is less frequent, particularly in the setting of broadly available rapid cryptococcal antigen tests.

The majority of the *Cryptococcus* capsule mass is constituted of glucoronoxylomannan and is generally known as cryptococcal antigen or CrAg [137]. Glucoronoxylomannan is produced by all *Cryptococcus* species. *Cryptococcus* spp. differ in the architecture on their capsular polysaccharides, and this may have consequences for development of diagnostic tests, possibly affecting glucoronoxylomannan detection.

There are many immunologic techniques for the diagnosis of cryptococcosis and innovative approaches have significantly diminished complexity and time of the test until results. Immunochemically based methods such as EIA, reverse passive latex agglutination, and immunochromatography are broadly used due to present advantages such as efficiency, easiness, and speed [138]. The main tools for the immunologic diagnosis of cryptococcosis are the tests aiming at CrAg in biological samples. The serum CrAg test is the most used noninvasive method to detect cryptococcal infection [139]. All these tests are able to diagnose disease caused by either *C. neoformans* or *C. gattii*.

### 6.1. Antibody Detection

Anti-*Cryptococcus* antibodies are usually not detectable during active cryptococcosis; therefore, antibody detection is not applied to the diagnosis of cryptococcosis. Tests based on antibody detection present highly variable results, depending on the type of assay. A study demonstrated that patients infected with *C. gattii* had a significantly higher prevalence of IgA and a non-significant higher prevalence of IgG compared to immunocompetent patients with *C. neoformans* infection [140].

### 6.2. Antigen Detection

An EIA, the PREMIER Cryptococcal Antigen Assay (Meridian Diagnostics, Inc.), was developed to detect cryptococcal capsular polysaccharide molecules in either serum or CSF specimens. This EIA does not need specimen pretreatment. The sensitivity with the serum samples is 100% and the specificity is 99%. Only the genus *Trichosporon*, which also produces glucoronoxylomannan-like molecules [141], caused a false positive reaction with this test [142].

Latex agglutination has been used for routine serological diagnosis, and some commercial kits are globally available to improve the diagnosis of cryptococcosis: CALAS Cryptococcal Antigen LA System (Meridian Bioscience Inc., Cincinnati, OH, USA), The Murex Cryptococcus Test (Remel), Crypto-LA test (Wampole Laboratories, Waltham, MA, USA) IMMY Latex-Crypto, and Pastorex Crypto Plus [143]. Usually, latex particles are linked to specific hyperimmune rabbit immunoglobulins, and are combined with different dilutions of clinical samples (CSF, sera, and urine) from cryptococcosis patients. A positive result at a 1:4 dilution clearly suggests *Cryptococcus* infection. Titers higher than 8 generally denote active disease. False positive results may be related to the presence of rheumatoid factor, which can be abolished after the biological sample treatment with pronase, dithiothreitol, or with boiling in EDTA. False positive results seldom occur when a glucoronoxylomannan-similar antigen is present in the clinical sample, for instance the polysaccharide of *Trichosporon* spp. The prozone-like effects due the excessive concentration of antigen or immune complexes can be abolished after clinical sample dilutions or treatment with pronase, respectively. The sensitivity of the latex agglutination is 94–100% and the specificity is 86–97% [144]. Performing the latex agglutination test requires a laboratory facility, and skilled laboratory workers, heat inactivation, and refrigeration of reagents [145].

A significant advance in testing for cryptococcal antigen was the development of an immunochromatographic assay, in a lateral flow assay format, the CrAg test (IMMY, Norman, OK, USA). This test identifies free capsular carbohydrates that have been released by *Cryptococcus* spp. into body fluids and addresses all pathogenic *Cryptococcus* species. The test is inexpensive and its sensitivity is equal to or higher than latex agglutination. The lateral flow assay detects the same antigen that is demonstrable by the commonly used latex agglutination and ELISA tests, and consequently, it has similar specificity. However, it is faster than the latex agglutination and ELISA, and produces clear results within 10 min. The test does not require electricity or advanced laboratory infrastructure, presents rapid turnaround time, and little technical knowledge is needed for development [146,147]. The CrAg lateral flow assay offers both qualitative and semi-quantitative results and can be used as a point-of-care assay for cryptococcosis diagnosis [148,149].

WHO published a guideline to diagnose and treat PLWHA infected by *Cryptococcus* spp. [150]. This guideline is centered on the fact that early diagnosis and treatment of cryptococcosis are essential to reduce mortality. The guideline emphasizes the value of a rapid CrAg test and pointed out that the CrAg test has high efficiency, is easy to perform, and is less dependent on laboratory expertise. This test would detect patients at high risk for cryptococcal disease and permit preemptive treatment with antifungals to avoid disease. This tactic is based on the fact that *Cryptococcus* antigenemia is noticeable around three weeks preceding the onset of clinical signs [151,152].

A number of other serologic procedures have been developed and are reported to improve cryptococcosis diagnosis. The alternative assays are based upon recombinant multi-epitope proteins, specific monoclonal antibodies, and the fungal heat shock protein 70 [153,154,155]. These tests have great potential to be inserted into the array of diagnostic tests.

## 7. Sporotrichosis

Sporotrichosis is a subcutaneous disease caused by human pathogenic fungi belonging to the genus *Sporothrix*, especially *Sporothrix schenckii*, *Sporothrix brasiliensis*, and *Sporothrix globosa*. The current major endemic areas of sporotrichosis include South America, especially Brazil; Asia, especially China and India; and Australia, but cases are also described in Europe and North America [4]. Since the 1970s, certain attempts were made to use immunologic tests as a tool for sporotrichosis diagnosis. For this mycosis, immunological tests are considered to be of inestimable diagnostic value, especially for extracutaneous and atypical forms of the disease [156].

### 7.1. Antibody Detection

Initially, immunoelectrophoresis, tube or latex agglutination, and immunodiffusion, using *Sporothrix* spp. antigens, were proposed for the serodiagnosis of sporotrichosis, but the efficacy of these methods was considered low, especially in cutaneous forms of the disease, which account for most cases of the mycosis [157,158,159]. Despite the low sensitivity, the immunodiffusion test is specific, without cross-reactions with cutaneous leishmaniasis or chromoblastomycosis, two infectious diseases with similar manifestations. Immunoelectrophoresis presents better sensitivity, with the presence of an anodic precipitation arc, called S arc, in reactive samples [157]. The sensitivity of agglutination tests was higher than that of precipitation tests, but only latex agglutination yielded satisfactory specificity results [158]. Due to this good specificity and sensitivity, latex agglutination is currently commercially available for sporotrichosis diagnosis (LA-*Sporothrix* antibody system—IMMY, Norman, OK, USA).

Because of the high endemicity of sporotrichosis in Brazil, noticed since the late 1990s, tests that are more sensitive for the cutaneous forms of the disease have been developed [160,161,162]. However, there is no consensus on the antigens used. ELISA has been performed with antigens obtained from the crude extract of yeast-like or filamentous forms of *Sporothrix*, besides purified antigens. The first described ELISA protocol for sporotrichosis diagnosis used a soluble antigen preparation from the *S. schenckii* yeast form. This antigen presented proteins ranging from 22 to 70 kDa and the whole performance of the assay showed 100% sensitivity and 90.5% specificity [163]. Later, the ELISA with a *S. schenckii* concanavalin-A binding antigenic fraction, isolated from the yeast cell wall, proved efficient in the detection of IgG antibodies, with a sensitivity of 90%, specificity of 80% and 86% global efficacy [160]. This ELISA can give results in a few hours and is very useful for therapeutic follow-up [164]. In addition, an ELISA with exoantigens produced by the filamentous form of *S. brasiliensis* was developed. The detection of IgG antibodies against these exoantigens resulted in 97% sensitivity and 89% specificity, with similar reactivity among patients with different clinical forms of the disease [161]. Moreover, IgA and IgM antibodies can also be evaluated using this method and a combination of IgG and IgA detection improves immunologic diagnosis, while the combination of IgG and IgM reactivities is suitable for therapeutic follow-up [165]. This ELISA protocol was also validated for diagnosis of sporotrichosis in cats, with better efficiency than the *S. schenckii* concanavalin-A binding antigenic fraction, as seen with human sera [166]. In addition, it was used to investigate an area supposedly without sporotrichosis endemicity, but with around 31% of positive samples from cats living in urban areas of the city [167]. Alvarado and collaborators developed an ELISA using a similar antigen produced by *S. schenckii*. Samples with immunologic reactivity were evaluated by immunodiffusion and counterimmunoelectrophoresis. They observed 100% of specificity and sensitivity superior to 98% with immunodiffusion, counterimmunoelectrophoresis, and ELISA [168]. Finally, an ELISA protocol using yeast cellular lysate proteins from *S. schenckii* was successfully used for a seroepidemiological survey in an endemic area in Brazil [169].

Western blot is less studied in the context of sporotrichosis diagnosis. The first protocol used the same soluble antigen from an *S. schenckii* strain described for the ELISA. All sera from patients with sporotrichosis presented reactivity against this antigen. Moreover, serum samples from individuals with extracutaneous manifestations of the disease reacted to more proteins than those from patients with cutaneous SPT: 15 to 20 and 8 to 10, respectively [163]. The other protocol uses a cell-free antigen preparation with the yeast-like form of *S. brasiliensis*, which presents up to 13 immunologic reactive bands, from 40 to 186 kDa. This Western blot showed 100% sensitivity, but just 50% specificity when an individual band was considered. Conversely, if only sera reactive to at least two distinct proteins are considered positive, sensitivity slightly decreases to 92.9% but specificity rises to 80% [170].

More recently, a lateral flow assay was developed to aid in the diagnosis of this mycosis. The test showed an accuracy of 82%, with sensitivity values dependent on sporotrichosis clinical forms. The sensitivity was greater for extracutaneous disease (92% sensitivity for ocular sporotrichosis) and lower for fixed-cutaneous sporotrichosis (78% sensitivity) [171].

An advance in the applicability of immunologic tests in sporotrichosis diagnosis is the possibility of analyzing different biological samples in addition to blood, such as CSF and synovial fluid. Furthermore, the serology is associated with an efficient clinical-serological correlation and cure control and can provide diagnosis even in immunocompromised patients [160,172,173,174].

### 7.2. Antigen Detection

The human pathogenic *Sporothrix* species produces an important antigen, rhamnomannan [164], that is not shared with other common mycoses agents, which usually produce galactomannan or glucuronoxylomannan. This would make antigen detection an interesting tool for sporotrichosis diagnosis. However, to the best of our knowledge, there are no immunological tests based on antigen detection for sporotrichosis diagnosis.

## 8. Talaromycosis

*Talaromyces marneffei* (formerly *Penicillium marneffei*) is a thermodimorphic fungal pathogen endemic in several countries of Southeast Asia, where it is a major threat to PLWHA. It was also reported in individuals traveling from several countries to an endemic area. There is a single report in Ghana of a patient who certainly did not travel to Southeast Asia [3]. Talaromycosis, the disease caused by *T. marneffei*, is a life-threatening infection with unspecific symptoms, which makes diagnosis difficult, especially in cases of imported disease [175].

The talaromycosis gold standard method is the culture of its agent from bone marrow, blood, sputum, and skin samples. The fungal filamentous form will develop at 25 to 30 °C, consisting of a white mycelium that turns green after sporulation with red diffusible pigment production. At 37 °C, yeast cerebriform or smooth colonies formed by cells that divide by binary fission will grow. Since rapid diagnosis is necessary, immunologic tests centered on antibody and antigen detection were developed to aid in this task [176].

### 8.1. Antibody Detection

The first immunologic test used for talaromycosis diagnosis was an immunodiffusion test using a concentrated filamentous secretome of the fungus grown for six weeks at 25 °C. Two or three precipitin bands are observed in this test against a rabbit hyperimmune serum [177]. However, this test presented low sensitivity and a single precipitin in positive samples when used for serum antibody detection in culture-proven talaromycosis patients, probably due to their immunosuppression [178].

To overcome this problem, an ELISA was developed using a recombinant 90 kDa mannoprotein of the fungus, Mp1p, expressed in *Escherichia coli*. This test was developed to be used in both immunocompetent and immunosuppressed patients, reaching an overall 82% sensitivity and 100% specificity, with around 80% positivity among PLWHA [179]. Later, the test with this mannoprotein was remodeled, now using *Pichia pastoris* to express the recombinant protein and a double-antigen sandwich ELISA format to detect anti-Mp1p antibodies. Again, 100% specificity was observed, but the sensitivity lowered to 13.3% [180].

The major limitation in employing antibody detection assays for talaromycosis diagnosis is their low sensitivity. In fact, these methods are highly specific, but culture, although time-consuming, has higher sensitivity [181].

### 8.2. Antigen Detection

As occurs with other endemic mycoses, antigen detection is particularly useful to diagnose talaromycosis in PLWHA that fail to produce specific *T. marneffei* antibodies. Special attention must be given to possible serologic cross-reactions that may occur when patients with talaromycosis are tested for *Histoplasma*, *Blastomyces*, or *Aspergillus* antigens [129,182].

Initially, antigen detection was performed using immunodiffusion, with better sensitivity values than antibody detection. In fact, a study with eight patients with proven talaromycosis revealed seven positive patients for antigen and two for antibodies (one patient was positive for both) using this method [178]. Afterwards, several methods for antigen detection were described, most of them in an ELISA format using polyclonal or monoclonal antibodies reactive to the Mp1p antigenemia or antigenuria. Their pooled sensitivity and specificity values, which have enrolled 320 *T. marneffei* infected patients and 1873 control individuals, are 82% and 99%, respectively [183]. The ELISA for Mp1p antigen detection is faster and more sensitive than traditional culture-based methods of diagnosis [184].

A dot-blot ELISA with a polyclonal anti-*T. marneffei* antibody coupled to FITC and an anti-FITC amplification system to detect antigen in urine samples presented 94.6% sensitivity and 97.3% specificity; however, these values were lower than those observed with the traditional ELISA format. The same reagents were used in a latex agglutination format and, with this method, 100% sensitivity was reached, with 99.3% specificity [185].

Lately, a lateral flow assay was created for talaromyces point-of-care diagnostics. This assay uses a monoclonal antibody reactive to a 50–180 kDa mannoprotein with a broad high molecular mass pattern conjugated with nanoparticles of colloidal gold for specific *T. marneffei* antigenuria detection. The detection limit is 3.12 µg/mL for *T. marneffei* antigen and sensitivity and specificity were 87.87% and 100%, respectively [176].

Some authors also report the combined detection of specific antigen and antibodies in patients with talaromyces. For instance, a study that used two ELISA formats to detect Mp1p, one with monoclonal antibody and the other with polyclonal antibody, and an ELISA to detect IgG anti-Mp1p presented 55%, 75%, and 30% sensitivity, respectively. However, the combined results of these tests yielded 100% sensitivity and 98% specificity [186]. Another study found 93.3% sensitivity when combining antigen and antibody detection results using a *P. pastoris* recombinant Mp1p in a sandwich ELISA [180].

Despite the high sensitivity and excellent specificity of the tests to detect *T. marneffei* antigens, no validation studies to endorse their use in the clinical setting exist. Moreover, there is a paucity of commercially available diagnostic kits for the serodiagnosis of talaromycosis [129].

## 9. Endemic Mycoses without Specific Immunologic Tests

### 9.1. Lacaziosis

The diagnosis of lacaziosis, a deep fungal infection caused by *Lacazia loboi*, is confirmed by clinical and histopathological methods. One study demonstrated that individuals with lacaziosis possess antibodies reactive to the gp43 antigen of *P. brasiliensis*, and also to a 193 kDa major *L. loboi* antigen through WB. The cross-reactivity occurs because, as supported by molecular studies, *L. loboi* and *P. brasiliensis* share a similar ancestor [187]. In contrast to prior reports, this study proposes that, during infection, *L. loboi* presents antigens that are distinct from that presented during paracoccidioidomycosis [188,189]. The molecular report of the 193 kDa molecule could generate precious data to comprehend the immunology of lacaziosis and its diagnostic applications, probably aiding in the management of infections caused by this resilient fungus.

### 9.2. Adiaspiromycosis

Adiaspiromycosis is a pulmonary infection of rodents, fossorial mammals, and their predators, infrequently occurring in humans, and is caused by the *Emmonsia crescens* and *Emmonsia parva*. During adiaspiromycosis, inhaled conidia enlarge to form non-replicating adiaspores. The infection usually involves the lungs, with rare cases of infection at other sites. Diagnosis is usually made by histopathology and the etiologic agent is identified based on the size of adiaspores [190]. There are a few immunologic tests to aid in adiaspiromycosis diagnosis; they are all designed to diagnose this mycosis in wild animals. Immunodiffusion and complement fixation showed good correlation with histopathology, with valuable sensitivity and specificity [191].

### 9.3. Emergomycosis

In the last few years, the emergence of emergomycosis, a rare, cosmopolitan fungal infection caused by the unusual dimorphic fungus *Emergomyces* spp. has been noticed among immunocompromised patients [192]. This mycosis also affects wild mammals. The main agents are *Emergomyces pasteuriana* (formerly *Emmosia pasteuriana*), *Emergomyces africanus*, *Emergomyces orientalis*, and *Emergomyces canadiensis* [193]. Multifocal pneumonia and cutaneous forms including papule-crusted injuries, nodules, wart-like lesions, or ulcerated plaques on the face, trunk, and extremities are the most common symptoms.

Diagnosis of emergomycosis remains challenging. Differential diagnosis includes HPM, BLM, tuberculosis, *Listeria* sp., and TLM. Among the endemic mycoses, emergomycosis should be considered in the histoplasmosis differential diagnosis since there is substantial clinical and histopathological findings overlapping between the two diseases [194]. The gold standard laboratory diagnosis is culture, with the growth, around 20–30 days, of white to beige, fastidious, and mycelial fungal colonies. Microscopic examination presents hyaline and thin hyphae with microconidia. However, potassium hydroxide slides of sputum or secretions are also helpful, where several small yeast cells around 1–3 µm in size are typically noticed. Histopathology displays an inflammatory, granulomatous process, with intra- and extra-cellular, 2–5 μm, round or ovoid yeast-like cells, alike in *H. capsulatum*, but *Emergomyces* have smaller and halo-less yeast cells [195].

There is no immunologic test or biomarkers with sufficient efficiency for emergomycosis diagnosis. Nevertheless, cross-reactivity has been seen with other dimorphic fungi. A retrospective case series was reported with two emergomycosis patients with a positive *Histoplasma* antigenuria, and one with positive 1,3-ß-D-glucan antigen detection [196]. Other reports also revealed cross-reactivity of the *Histoplasma* galactomannan in urine samples of patients infected with *E. africanus*. A commercial *Histoplasma* EIA had suitable accuracy to diagnose proven histoplasmosis, but cross-reactions were seen in urine samples from individuals with invasive infections due to *E. africanus* and in culture filtrates of this species and other related fungal pathogens [197]. *Emergomyces* spp. may present cross-reactivity with *Histoplasma* antigenuria assays, but a negative result cannot reject diagnosis [198]. Clinical research main concerns must incorporate the validation of existing and new diagnostic tests to improve comprehension of emergomycosis epidemiology, to aid in diagnosis, and to feasibly identify individuals who may benefit from preemptive therapeutics.

## 10. Conclusions

Endemic mycoses result from infection mainly due to dimorphic fungi, and continue to cause substantial morbidity and mortality, especially in selected regions. The diagnosis of endemic mycoses is typically achieved by an association of clinical, epidemiological, and laboratory information. Substantial progress has been made in non-culture-based methods to diagnose these mycoses with the development of a variety of techniques for the detection of antibodies (Table 2), antigens (Table 3), and nucleic acids. The serological methods described for the diagnosis of endemic mycoses have their strengths and weaknesses and demand critical evaluation by mycologists and medical doctors. Nevertheless, not all tests herein described are entirely available across the world, which complicates the competence to diagnose and treat patients with endemic mycoses. Moreover, the immunological status of the patient and manifestation of these diseases influence the efficacy of the diagnostic test. Continuing efforts to improve or develop diagnostic tests will facilitate our diagnostic capacity. However, such assays will require validation in populations from diverse regions of the world prior to their general application in routine diagnosis. Results obtained from a panel of serologic diagnostic tests play an important role in the diagnosis of endemic mycoses, allowing more rapid and precise diagnosis, which would lead to earlier treatment. However, the gold standard for diagnosis continues to be the culture, and the correlation between molecular data and phenotypic characteristics is crucial in identifying the etiological agents of endemic mycoses.

## Figures and Tables

**Table 1:** Immunologic methods used for antigen or antibody detection for the diagnosis of the major endemic mycoses.

Method	BLM	CDM	HPM	PCM	CRY	SPT	TLM
Complement fixation	Ab	Ab	Ab	Ab	-	-	-
Immunodiffusion	Ab	Ab/Ag	Ab	Ab	-	Ab	Ab/Ag
Counterimmunoelectrophoresis	-	-	-	Ab/Ag	-	Ab	-
Tube precipitin	-	Ab	-	-	-	Ab	-
Latex agglutination	-	Ab	Ab	Ab	Ag	Ab	-
Lateral flow assay	-	Ab	Ag	-	Ag	Ab	Ag
ELISA	Ab/Ag	Ab/Ag	Ab/Ag	Ab/Ag	Ab/Ag	Ab	Ab/Ag
Western blot	-	Ab	Ab	Ab/Ag	-	Ab	-
Radioimmunoassay	Ab	-	Ag	Ag	-	-	-

BLM: blastomycosis; CDM: coccidioidomycosis; HPM: histoplasmosis; PCM: paracoccidioidomycosis; CRY: cryptococcosis; SPT: sporotrichosis; TLM: talaromycosis; Ab: antibody; Ag: antigen; ELISA: enzyme-linked immunosorbent assay.

**Table 2:** Summary of sensitivity and specificity values of immunological tests used for diagnosis of endemic mycoses by antibody detection.

Disease	Test	Sensitivity	Specificity	References
Histoplasmosis	ID	75–95	100	[58]
	CF	72–95	70–80	[58]
	EIA	66–97	54–100	[65,67,68,69,70]
	Western blot	95	94	[72,73]
Paracoccidioidomycosis	ID	17–100	43.3–100	[99,103,104,110,122]
	EIA	75–100	100	[103,115,116]
	Western blot	77.3–100	73.3–100	[114,118,119,120,123]
	CIE	95–100	100	[99,103,110]
Blastomycosis	ID	28	100	[6]
	CF	9	100	[6]
	EIA	77	92	[6]
Coccidioidomycosis	ID	50–90		[6,44]
	CF	67–75		[6]
	EIA	54–92	97	[6,44]
Sporotrichosis	ID	98	100	[168]
	CIE	98	100	[168]
	EIA	90–100	80–100	[160,161,163,168]
	Western blot	93–100	50–80	[163,170]
Talaromycosis	ID	25	NE	[177,178]
	EIA	13–82	81–100	[179,180]

ID: immunodiffusion; CF: complement fixation; EIA: enzyme immunoassay; CIE: counterimmunoelectrophoresis.

**Table 3:** Summary of sensitivity and specificity values of immunological tests used for diagnosis of endemic mycoses by antigen detection.

Disease	Test	Target	Specimen	Sensitivity	Specificity	References
Histoplasmosis	RIA	100 kDa (HPA)	Urine	96.7	100	[76]
			Serum	78.7	100	
	EIA	69–70 kDa	Serum	71.4	85.4	[80]
	EIA	Galactomannan	Urine	61.9–100	32–99.8	[82,83,84,85,86,87]
			Serum	92.3	99	[83]
			BAL	93.5	97.8	[84]
	EIA	Cell wall antigen	Serum	81	95	[79]
	EIA	100 kDa (HPA)	Urine	86	94	[81]
	LFA	Galactomannan	Urine	96	96	[88,89]
			Serum	92	94	
Blastomycosis	EIA	Galactomannan	Urine	76–90	NE	[24]
			Serum	52–82	NE	
Coccidioidomycosis	EIA	Chitinase-1	Serum	87	97	[50]
Paracoccidioidomycosis	EIA	gP43 glycoprotein	Serum	95.1	97.5	[131]
	EIA	*P. brasiliensis* total and filtrate antigen	Urine	75	100	[132]
	EIA	87 kDa	Serum	80.4	81.4	[130]
Talaromycosis	ID	*T. marneffei* yeast secretome	Serum	58.8	100	[176]
		*T. marneffei* filamentous secretome	Serum	87.5	NE	[176,178]
	LA	*T. marneffei* yeast secretome	Serum	76.5	100	[176]
		Whole-fission-form yeast of *T. marneffei*	Urine	100	99.3	[176,185]
	Dot-blot	Whole-fission-form yeast of *T. marneffei*	Urine	94.6	97.3	[176,185]
	EIA	Whole-fission-form yeast of *T. marneffei*	Urine	97.3	98	[176,185]
	EIA	Yeast and mycelial antigens	Serum	72–92.5	97.5–100	[176]
	EIA	Mp1p	Serum	55–75	99.4–99.6	[176,179,186]
	EIA	TM cytoplasmic yeast antigen	Serum	100	100	[176]
	LFA	TM cytoplasmic yeast antigen	Urine	87.9	100	[176]
Cryptococcosis	LA	Cryptococcal capsular antigen	Serum, CSF	94–100	86–97	[144]
	EIA	Cryptococcal capsular antigen	Serum, CSF	99	97	[142]
	LFA	Cryptococcal capsular antigen	Serum, CSF	100	97–99	[149]

RIA: radioimmunoassay; EIA: enzyme immunoassay; LFA: lateral flow assay; LA: latex agglutination; ID: immunodiffusion; CSF: cerebrospinal fluid.

## Data Availability

Not applicable.
